# Serotonin Receptor B May Lock the Gate of PTTH Release/Synthesis in the Chinese Silk Moth, *Antheraea pernyi*; A Diapause Initiation/Maintenance Mechanism?

**DOI:** 10.1371/journal.pone.0079381

**Published:** 2013-11-04

**Authors:** Qiushi Wang, Ahmed A. M. Mohamed, Makio Takeda

**Affiliations:** Graduate School of Agricultural Science, Kobe University, Kobe, Japan; Swedish University of Agricultural Sciences, Sweden

## Abstract

The release of prothoracicotropic hormone, PTTH, or its blockade is the major endocrine switch regulating the developmental channel either to metamorphosis or to pupal diapause in the Chinese silk moth, *Antheraea pernyi*. We have cloned cDNAs encoding two types of serotonin receptors (5HTR_A and B_). 5HTR_A_-, and 5HTR_B_-like immunohistochemical reactivities (-ir) were colocalized with PTTH-ir in two pairs of neurosecretory cells at the dorsolateral region of the protocerebrum (DL). Therefore, the causal involvement of these receptors was suspected in PTTH release/synthesis. The level of mRNA^5HTRB^ responded to 10 cycles of long-day activation, falling to 40% of the original level before activation, while that of 5HTR_A_ was not affected by long-day activation. Under LD 16:8 and 12:12, the injection of dsRNA^5HTRB^ resulted in early diapause termination, whereas that of dsRNA^5HTRA^ did not affect the rate of diapause termination. The injection of dsRNA^5HTRB^ induced PTTH accumulation, indicating that 5HTR_B_ binding suppresses PTTH synthesis also. This conclusion was supported pharmacologically; the injection of luzindole, a melatonin receptor antagonist, plus 5th inhibited photoperiodic activation under LD 16:8, while that of 5,7-DHT, induced emergence in a dose dependent fashion under LD 12:12. The results suggest that 5HTR_B_ may lock the PTTH release/synthesis, maintaining diapause. This could also work as diapause induction mechanism.

## Introduction

Many living organisms can monitor day or night length to adjust their behavior, metabolism, physiology and developmental course to maximally adapt for an adverse or favorable season. This is called photoperiodism, which remains as a biological mystery, at least at the molecular level. This system is complex, consisting of several functional subunits; a photoreceptor, a clock/timer, a summation mechanism counting effective photoperiodic cycles and an endocrine switch. The photoperiodism of insects, poikilotherms with wide distributions and short life, shows overwhelming sophistication [1,2]. It is important to understand the photoperiodic mechanism and its effects on the seasonal demography of pest insects from the pest management point of view, as well as scientific curiosity. Therefore, many scientists have attempted to elucidate this mechanism. However, the molecular mechanism still remains obscure and dispute over the mode of photoperiodic time measurement continues; hourglass timer vs. circadian clock [3,4]. 

We chose *Antheraea pernyi* as a model animal to study this issue, since this is a classical organism used for the elucidation of the endocrine mechanism for metamorphosis and pupal diapause [5]. Other advantages of using this species include the availability of circadian clock genes and the prothoracicotropic hormone (PTTH) gene [6]. *A. pernyi* enters pupal diapause when raised under short days, but diapause is averted under long days. Photoperiod affects the release of PTTH. When it is released, diapause is terminated or averted, and when it is not released, diapause results or is maintained. Diapause is also terminated after long storage at a low temperature [7]. However, the question of what releases PTTH or conversely what stops its release remains to be answered. We have monitored brain neurotransmitter dynamics and enzymatic activity changes during diapause and photoperiodic activation [7,8]. 

Sauman and Reppert [6] have shown the juxtaposion of P_ER_ (PERIOD)-ir to PTTH-ir in *A. pernyi* and Ichihara [9] have demonstrated the colocalization of DBT-, NAT-, HIOMT-, and melatonin-ir with P_ER_-ir. We continuted to carry out immunohistochemical localization of circadian clock proteins, neurotransmitter receptors, neuropeptides and neurotransmitter metabolic enzyme-like antigens, here showing the colocalization of Cyc- and Clk-ir with P_ER_-ir. The results suggest that the indolamine metabolic pathway may mediate circadian output pathway to PTTH release.

RIA showed that “immunoreactive melatonin” increased in the brain and hemolymph of diapause pupa of *A. pernyi* under long-day condition and REA, redioengymatic assay, showed that this activation was caused by the increased insect anylalkylamine NAT (iaaNAT, aaNAT, NAT). We have retrieved cDNA encoding NAT from *A. pernyi* on the PCR-based cloning and show enzymatic activity of baculovirus expressed protein with serotonin (5-hydroxytryptamine, 5HT) as a substrate [10]. These results suggest that melatonin stimulates PTTH release and the mechanism that dictates circadian output involves the aaNAT gene [11]. The injection of dsRNA^aaNAT^ abolished photoperiodism under LD 16:8. The upstream promotor region of this NAT contained multiple E-boxes and melatonin receptor (MT), MT-ir was observed in PTTH neurons (unpublished data). During this course of study, we noticed not only MT-ir but also serotonin receptors (5HTRs)-ir in PTTH-ir cells. The neurosecretory cells (ns cells) secreting PTTH were located in the dorsolateral protocerebrum (DL) of *A. pernyi* [6], and this condition was also found in *Bombyx mori* and *Manduca sexta* [12,13]. cDNAs encoding PTTH from *B. mori*, *M. sexta* and *A. pernyi* were successfully cloned and sequenced [14,15,6]. In *A. pernyi*, PTTH release was a gated phenomenon under control of the circadian clock that terminates pupal diapause under long-day conditions [4,16,5]. The release of PTTH is also under the regulation of the photoperiodic/circadian clock, and in *Periplaneta americana* melatonin stimulates PTTH release and serotonin suppresses it [17]. In *B. mori*, serotonin stimulates PTTH release [18] but serotonin is an upstream precursor for melatonin. Therefore it cannot be determined which of the two indolamines is the direct releaser of PTTH. The question to be asked here is whether diapause is simply a default condition for melatonin activation mechanism or it requires a special mechanism of developmental arrest. If the former is the case, these were no need of 5HTR in PTTH neurons.

5HT is a major biogenic amine distributed in the insect central nervous system [19]. 5HT regulates behaviors such as mood, sleep, memory and sex in humans [20]. It also plays important roles in the circadian system of insects [21]. Recently, studies on 5HTRs have progressed in insects, especially in Lepidoptera and Diptera [22]. 5HTRs are now classified into 7 subfamilies in insects [23]. The honeybee is known to express four 5HTR subtypes (*Am*5HT_1A_, *Am*5HT_1B_, *Am*5HT_2_ and *Am*5HT_7_). Hiragaki et al. [24] have cloned two putative 5HTR subtypes from the brain of *A. pernyi* (*AP*5HTR_A_ and *AP*5HTR_B_). However, the roles of these 5HTRs in the regulation of diapause are still unclear. This is the focus of this investigation.

## Materials and Methods

### Ethics Statement

This study was carried out in strict accordance with the recommendations in the Guide for the Care and Use of Laboratory Animals of Kobe University. The protocol was approved by the Committee on the Ethics of Animal Experiments of Kobe University (Permit Number: 19-5-01). All surgery on rabbits was performed under sodium pentobarbital anesthesia.

### Insects

Diapause pupae of a univoltine strain of *A. pernyi* were either shipped or personally carried by researchers from Henang Province, to Japan. The diapause pupae were stored under LD 12:12 at 25°C for 2 weeks. Diapause pupae were used for physiological experiments within 4 months, during which time photoperiodism was securely maintained.

### Primary antibodies

Antibodies against two *Ap*5HT receptors, 5HTR_A_ and _B_, were raised by injecting totally four New Zealand white rabbits with synthetic peptides conjugated with KLH. The 18-amino-acid peptide from 447 to 464 of the deduced sequence of *A. pernyi* 5HTR_A_ and another peptide corresponding to 20 amino acids from 429 to 448 of the deduced sequence of *A. pernyi* 5HTR_B_ were used as antigens. Immunizations were performed using two groups rabbits (n=2 for each group). The antigens and TiterMax Gold were mixed at a ratio of 1:1 (v:v) before injection. Blood samples of 10 mL were harvested from ear vein, antibody detection was analyzed from 2 weeks to 4 weeks. The whole blood collected during general anesthesia by using sodium pentobarbital. Their specificities and details of the antibody have been described previously in Shao et al [25]. The two sequences have no overlap. A kind gift from Drs. Ivo Sauman of the Czech Academy of Sciences, Ceske Budejovice and Steven Reppert of antiserum against *A. pernyi* PTTH (*Ap*PTTH) raised in rabbit (residues 132-152; GenBank accession no. AAB05259) was used. We raised antibodies against *B. mori* PTTH (*Bm*PTTH) in rat (antigen sequence: GNIQVENQAIPDPPCTCKYKK) (Genmed, Taxas, USA). This antibody was also used for double-staining and confirmation. 

### Immunohistochemistry

Immunohistochemistry was performed on the BR-SOG of male and female adults and pupae of *A. pernyi*. Dissection was conducted during the daytime from pupae 5 days after the activation by LD 16:8. The BR-SOG, frontal ganglion (FG), corpora cardiaca (CC) and corpora allata (CA) were dissected from the water-anesthetized animals in sterile saline. The tissues were fixed overnight at 4°C in Bouin solution. Standard histochemical methods were used for tissue dehydration, embedding in paraffin, sectioning (8 µm), deparaffinization and rehydration according to a previous report [9]. The sections were blocked with 1.5% normal goat serum diluted in Tris-buffered saline (TBS; 135 mM NaCl, 2.6 mM KCl, 25 mM Tris-HCl, pH7.6) for 30 min at room temperature (RT). Subsequent overnight incubation with primary antibodies (Table S1) diluted with blocking serum was conducted in a humidified chamber at 4°C. In the controls, the primary antibodies were replaced with normal serum. After 3 rinses with TBS, each for 10 min, the sections were incubated for 90 min with a biotinylated secondary antibody, rinsed 3 times for 10 min with TBS and treated for 30 min with VECTASTAIN ABC kit (Vector Laboratories, Burlingame, CA, USA). Following 3 rinses each for 10 min and one with 0.1 M Tris-HCl, pH7.5 (5 min), the HRP enzymatic reaction was visualized with hydrogen peroxide (0.005%) and 3,3’-diaminobenzidine tetrahydrochloride (DAB, 0.25 mM in 0.1 M Tris-HCl, pH7.5). Stained sections were dehydrated and mounted on Bioleit mounting medium (Kouken Rika, Osaka, Japan). The mounted specimens were examined under a BX50F4 microscope (Olympus, Tokyo, Japan). 

For the double labeling (with antibodies derived from the same animal), experiments were performed according to the method of Hiragaki et al. [26]. Anti-*Ap*5HTR_A/B_ antibody (Table S1) was incubated overnight at 4°C. After rinsing (3×) with TBS-Tw, the slides were incubated with secondary antibody for 60 min. After rinsing (3×) with TBS-Tw, they were treated for 30 min with VECTASTAIN ABC reagent. Then, sections were treated with TSA Biotin System (Perkin Elmer, MA, US), which can induce covalent bonds between tissue and biotin on the position of first color. Anti-*Ap*5HTR_A/B_ antibody was stripped out of the sections for 24 hours at RT in stripping buffer (100 mM 2-mercaptoethanol, 50 mM glycine-HCl, pH2.2) in a 40 V horizontal electric field. The sections were then incubated with anti-*Ap*PTTH (Table S1) overnight at 4°C. The slides were treated for 30 min with VECTASTAIN ABC reagent. After rinsing (3×) with TBS-Tw, the slides were incubated with Alexa Fluor 488-conjugated (green) goat anti-rabbit IgG for 60 min at RT. After rinsing (3×) with TBS-Tw, the biotin signal was visualized with green fluorophore using a TSA Labeling Kit #42. Finally, the slides were rinsed (3×) with TBS-Tw, mounted in Aqua Ploymount and observed using a BX50F4 microscope (Olympus, Japan). 

For double labeling (with antibodies derived from different animals), we used a combination of anti-*Ap*5HTR_A/B_ (Antibody 2) with anti-*Bm*EH (Antibody 1) or with anti-*Bm*PTTH (Antibody 1), as follows. Drop cocktail of both primary antibodies (Table S1) diluted in TBS-Tw containing 1% BSA was used to incubate the sections overnight at 4°C. After rinsing (3×) with TBS-Tw, the slides were incubated with horse anti-goat IgG (H+L)-biotin or goat anti-mouse IgG (H+L)-biotin (Vector Laboratories, CA, US) for 1.5 hours. After rinsing (3×) with TBS-Tw, the slides were incubated with Alexa Fluor 488-conjugated goat anti-rabbit IgG for 60 min at RT (Invitrogen, Tokyo, Japan). After rinsing (3×) with TBS-Tw, the biotin signal was visualized with red fluorophore using TSA Labeling Kit #42 with Alexa Fluor 555. Finally, the slides were rinsed (3×) with TBS-Tw, mounted in Aqua Ploymount and observed using a BX50F4 microscope (Olympus, Japan). 

In control experiments, the primary antibodies were replaced with normal goat serum or rat serum. As an additional control for binding specificity, the *Bm*PTTH anti-bodies were pre-incubated with a 100 molar excess of antigen before immunological staining. In both cases, no staining was observed above background.

### RNA extraction and cDNA synthesis

The BR-SOG of *A. pernyi* was dissected and immediately transferred to liquid nitrogen and total RNA was isolated by using the RNAiso Plus reagent (Takara, Japan). Five hundred nanograms of total RNA with primers using ReverTra Ace kit (Toyobo Co. Ltd., Osaka, Japan) was used for synthesizing the cDNA.

### Preparation and injection of dsRNA

PCR products of 539 bp for 5HTR_A_ (accession number EU402612.1) and 345 bp for 5HTR_B_ (accession number EU402613.1) were prepared by gene-specific primers (5HTR_A_-T7-F, 5HTR_A_-T7-R and 5HTR_B_-T7-F, 5HTR_B_-T7-R) (Table 1) in which the T7 promoter was attached to the 5’ end of each primer. dsRNAs were synthesized after incubation of the purified PCR product at 37°C for 4 hours with MEGAscript RNAi kit (Ambion, CA, USA) according to the manufacturer’s instructions. The control dsRNA was generated from the GFP gene of jellyfish (dsRNA^GFP^) that should have no effect on the target gene [27]. The dsRNA and Metafectene PRO (Biontex, Planegg, Germany) were mixed at a ratio of 1:1 (v:v) before injection. One μg of dsRNA was injected into individual pupae. 

**Table 1:** A list of primers used in the experiments.

Name	Sequence of the primers
5HTR_A_-T7-F	TAATACGACTCACTATAGGGAGAAATACCTCCCGACTGTGTAAATATG
5HTR_A_-T7-R	TAATACGACTCACTATAGGGAGACGTAATGTCACTTAAACAACAGGTG
5HTR_A_-F	GATAGTTGACGGTAAAATCGTCGT
5HTR_A_-R	AGCAGTTCCCGTCGTACCAG
5HTR_B_-T7-F	TAATACGACTCACTATAGGGAGAATCAGAGGATCTAAGATGTGTCGTC
5HTR_B_-T7-R	TAATACGACTCACTATAGGGAGACTAGATTGTTTTTCCGGGCTAGTAT
5HTR_B_-F	TATGGCTAGGTTACTTCAACTCCAC
5HTR_B_-R	GTTTCAAACTAGACGAGGTCAGTCA
GFP-T7-F	TAATACGACTCACTATAGGGAGACCTGAAGTTCATCTGCACCAC
GFP-T7-R	TAATACGACTCACTATAGGGAGAACGAACTCCAGCAGGACCAT
RP49-F	AAGACCCGTCACATGCTACC
RP49-R	GCGTTCGACGATTAACTTCC

Underlined sequences are the T7 promoter and the highlighted sequences are the original primers.

### qRT-PCR

The qRT-PCR was performed with the SYBR^®^ Green and THUNDERBIRD^TM^ qPCR Mix (Toyobo Co. Ltd., Osaka, Japan), with the forward and reverse primers designed [Table 1]. Cycling parameters were 95°C for 1 min to activate DNA polymerase, and then 40 cycles of the following PCR amplification with primers were performed using the following temperature program; 95°C for 15 sec and 60°C for 1 min. To confirm the specificity of the PCR products, melting curves were determined using the software ABI 7000 Sequence Detection System (Applied Biosystems, Foster City, CA, USA). Amounts of amplified products were calculated from cDNA standard curves generated for each PCR run. For expression levels of each transcript, the *rp49* (accession number DQ296005.1) mRNA was used as the internal control. For each gene, the primers used in qRT-PCR (Table 1) were designed outside the region of knocking down for RNAi. The sizes of the PCR products were 180 bp for 5HTR_A_ and 174 bp for 5HTR_B_. 

### SDS-PAGE and western blotting analysis

Pupal BR-SOGs of *A. pernyi* under LD 16:8 were collected 72 h after injection of nuclease-free water (control), dsRNA^GFP^ or dsRNA^5HTRB^. The samples were homogenized in 200 µl of sample buffer (25% 0.5 M Tris-HCl, pH 6.8, 4% SDS, 20% glycerol, 20% 2-mercaptoethanol, 0.08% bromophenol blue) using Branson Sonic Power (CT 06810). The homogenate was centrifuged (10,000 *g*, 5 min at 4°C) to eliminate the cuticle and cell debris, from that the supernatant was collected and denatured at 95°C for 10 min before storage at -20°C until use. Fifteen µl sample was loaded per lane on 10% SDS-polyacrylamide, and SeeBlue Plus2 Pre-stained Standard marker 4-250 kDa (Invitrogen, USA) was used to estimate the molecular size of separated proteins. The proteins were transferred onto a PVDF membrane (GE Healthcare Bio-Science Co., Piscataway, NJ, USA). The membrane was treated with commercial blocking solution (Blocking One, Nacalai Tesque, Japan) for 30 min at room temperature. The membrane was incubated with primary antibodies for *Ap*PTTH (1:10,000) and *Bm*EH (1:20,000) overnight at 4°C, followed by the corresponding HRP-conjugated secondary antibody for 1 h at room temperature. The immunoreaction was visualized using an ECL system. The image analysis software of Image J was used to determine the densities of specific bands.

#### 5HT, luzindole and 5,7-dihydroxytryptamine (5,7-DHT) injections

Five pmoles 5HT in 5 µl of distilled water (D.W.) and 5 pmoles luzindole (TocRis, USA) in 5 µl of DMSO were injected using a Hamilton syringe (Hamilton Company, USA) into the intersegmental membrane between the thorax and the abdomen for each pupa. The control was injected with 5 µl of D.W. and 5 µl of DMSO into each pupa under LD 16:8.

0.1, 1 and 10 pmoles of 5,7-DHT (Sigma, USA) in 10 µl of D.W. were injected by using a syringe into each pupa as mentioned above. The same volume of D.W. was injected into each pupa as a control group.

### Statistical analysis

The results are expressed as mean ± S.E.M. p<0.05 was considered the level of significant difference between means by one-way ANOVA (Fishers, LSD) and Kaplan-Meier.

## Results

### mRNA level of 5HTRs and responsiveness to long day exposure

**Figure 1:**
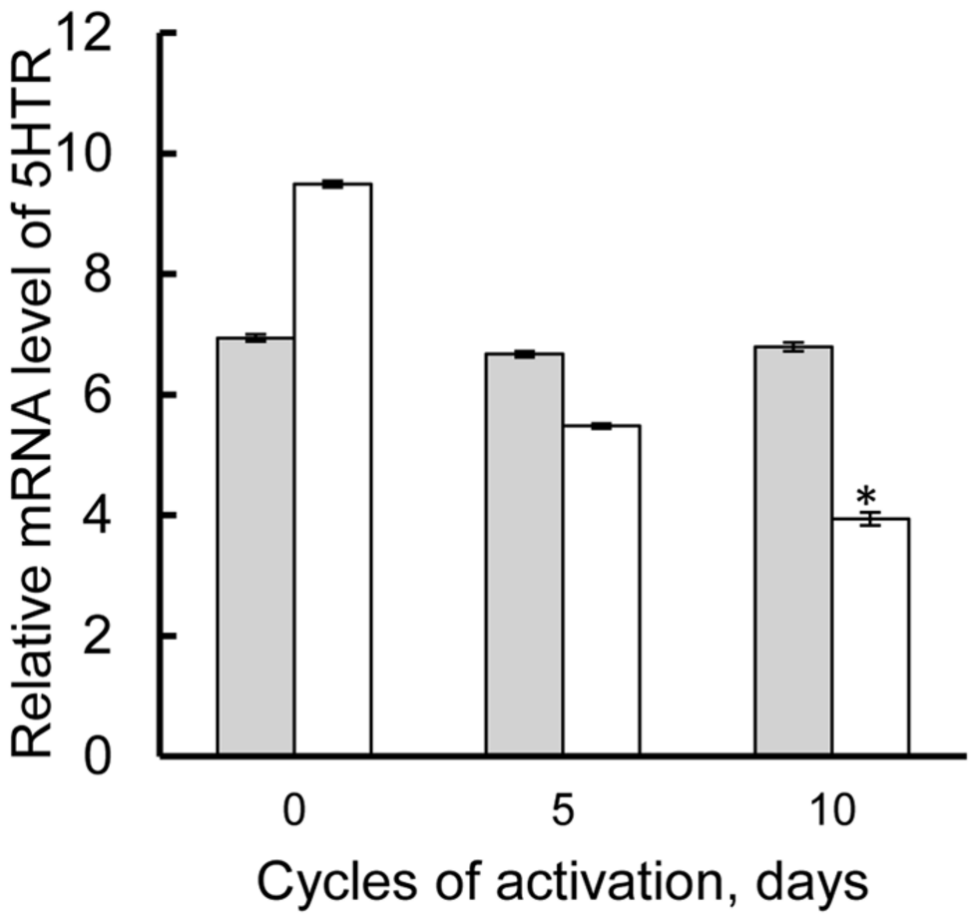
Relative mRNA levels of 5HTRs upon long-day activation. Diapause pupae were exposed to LD 16:8 for 0, 5 and 10 cycles at 25°C and mRNA level of 5HTR_A_ (gray bar) and 5HTR_B_ (white bar) in the BR-SOG was determined by real time PCR. The results are presented as the mean ± S.E.M. from three independent experiments. Asterisks indicate significant difference from 0-day incubation by one-way ANOVA (Fisher's, LSD). *p<*0.05.

To determine the proximity of 5HTRs to photoperiodic mechanisms, we exposed diapause pupae to LD 16:8 for 0, 5 and 10 days quantifying the mRNA levels. The relative level of 5HTR_B_ mRNA after 10 days of incubation under LD 16:8 was significantly lower than the other treatments. However, that of 5HTR_A_ mRNA was almost constant among the treatments (Figure 1). Only 5HTR_B_ responded to photoperiodic activation.

### Co-localization of PTTH-ir and EH-ir with 5HTR-ir in adult and pupal BR-SOG of *A. pernyi*

Next, we localized these receptors immunohistochemically. Data on antibodies against *Ap*5HTRs, *Ap*/*Bm*PTTH and *Bm*EH are listed in Table S1. The antibodies recognized several immunoreactive neurons in the brain of less than one day-old adult and early pupa of *A. pernyi*. Tables 2 and 3 summarize the loci and numbers of those neurons. 

**Table 2:** The number of immunopositive cells in both hemispheres in the cephalic ganglia of female adult *A. pernyi* less than 24 hours after emergence and intensity of their immunostaining.

Antigen targeted	Locus			
	DL	PI	DC	SOG	
*Ap*5HTR_A_	4++++	-	-	-	
*Ap*5HTR_B_	4+++	-	-	2+++	
*Ap*PTTH	4++++	-	-	-	
*Bm*PTTH	4++++	-	-	-	
*Bm*EH	-	-	4++++	2++++	

PI: pars intercerebralis; DL: dorsolateral region of the protocerebrum; DC: deutocerebrum; SOG: subesophageal ganglion. Immunoreactivity was quantified as strong (++++) , considerable (+++) , moderate (++), weak (+), and negative (-).

**Table 3:** The number of immunopositive cells in both hemispheres in the cephalic ganglia of 5 days after the exposure to LD 16:8 of *A. pernyi* and intensity of their immunostaining.

Antigen targeted	Locus			
	DL	PI	DC	SOG	
*Ap*5HTR_A_	4++++	-	-	-	
*Ap*5HTR_B_	4++	-	2+	-	
*Ap*PTTH	4++++	-	-	-	
*Bm*PTTH	4++++	-	-	-	
*Bm*EH	-	2++	2++++	-	

PI: pars intercerebralis; DL: dorsolateral region of the protocerebrum; DC: deutocerebrum; SOG: subesophageal ganglion. Immunoreactivity was quantified as strong (++++) , considerable (+++) , moderate (++), weak (+), and negative (-).

**Figure 2:**
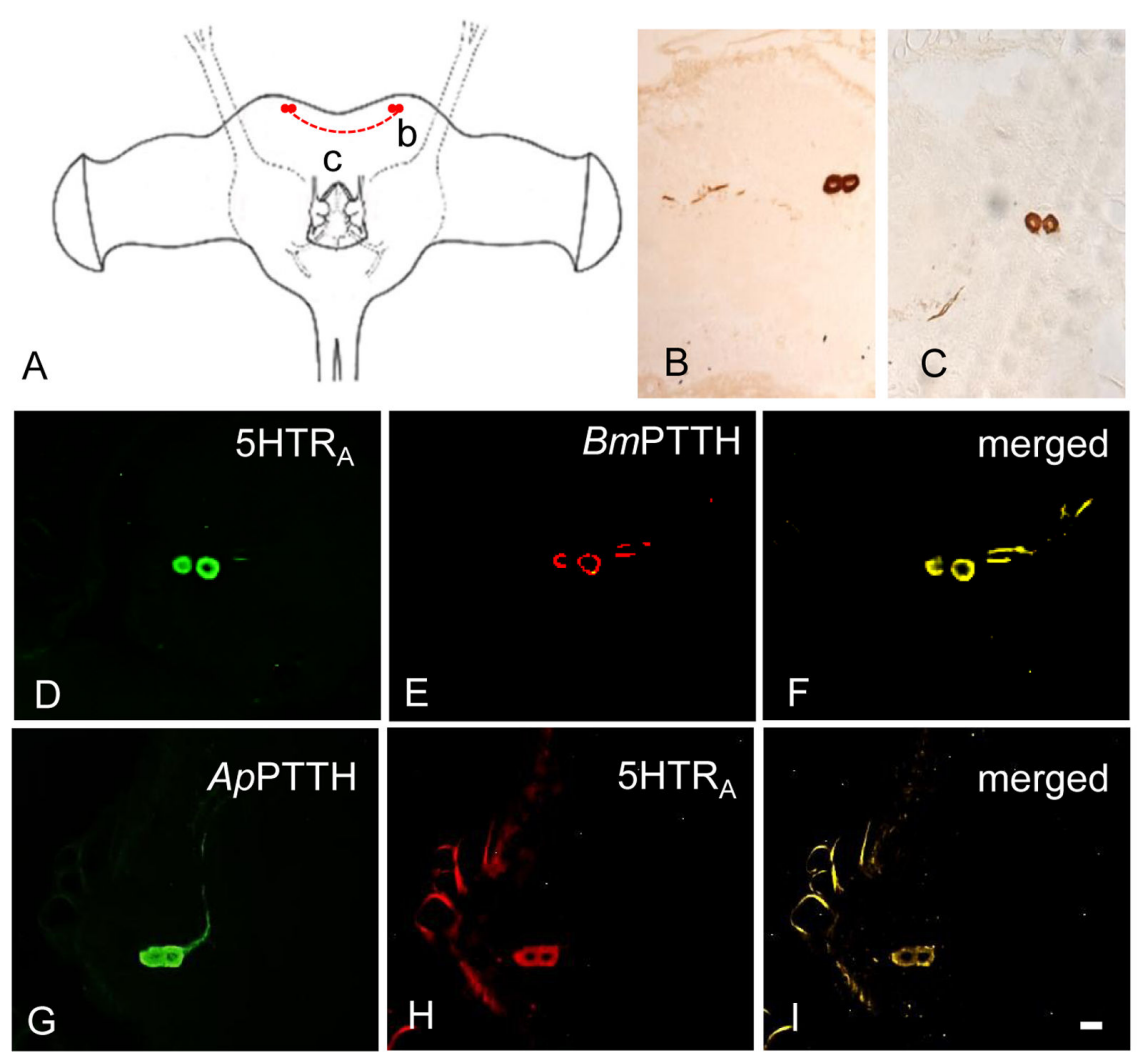
Colocalization of 5HTR_A_- and PTTH-ir in the adult BR-SOG of ***A. pernyi***. *Bm*PTTH-ir/*Ap*PTTH-ir in the BR-SOG of female adult with 24hrs after emergence was co-localized with *Ap*5HTR_A_-ir. (A) The locations of detected cells. Lower-case letters correspond to the regions (capital letters) shown in the photographs (e.g., b to B). (B) Two large PTTH-ir neurons in the DL region. (C, D, H) Two large 5HTR_A_-ir neurons in the DL region. (E) *Bm*PTTH-ir in the DL region. (F) Merged image of *Bm*PTTH- and 5HTR_A_-ir in the DL region. (G) *Ap*PTTH-ir in the DL region. (I) Merged image of *Ap*PTTH and 5HTR_A_ in the DL region. Scale bar = 100 µm.

**Figure 3:**
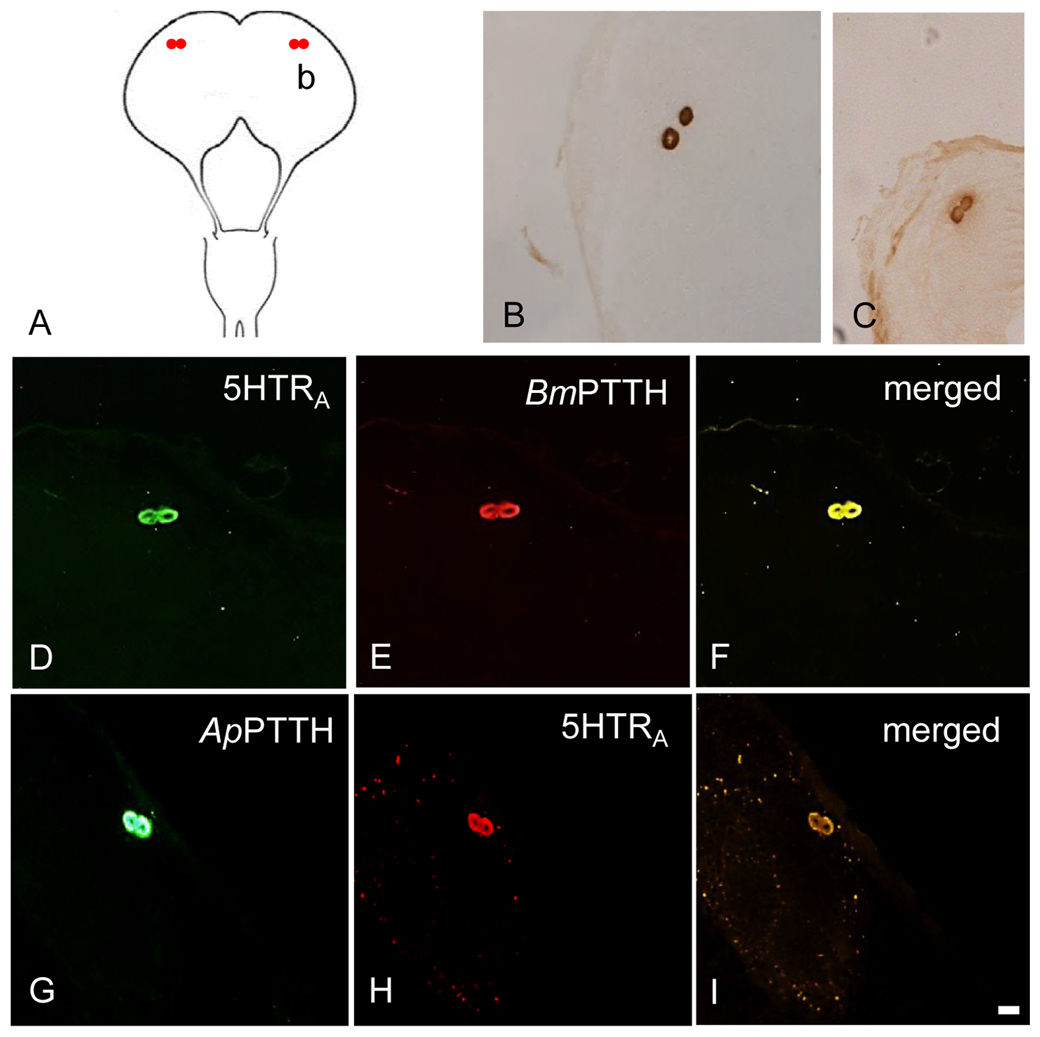
Colocalization of 5HTR_A_- and PTTH-ir in the early pupal BR-SOG of ***A. pernyi***. *Bm*PTTH-ir/*Ap*PTTH-ir was co-localized with *Ap*5HTR_A_-ir in the BR-SOG of 5-day-old pupa. (A) The location of detected cells. Lower-case letters correspond to the regions shown in the photographs (e.g., b to B). (B) Two large PTTH-ir neurons in the DL region. (C, D, H) Two large 5HTR_A_-ir neurons in the DL region. (E) *Bm*PTTH-ir in the DL region. (F) Merged image of *Bm*PTTH- and 5HTR_A-_ir in the DL region. (G) *Ap*PTTH-ir in the DL region. (I) Merged image of *Ap*PTTH-ir and 5HTR_A-_ir in the DL region. Scale bar = 100 µm.

A pair of large neurosecretory (ns) cells showed *Ap*PTTH-ir in the dorsolateral (DL) region of protocerebrum of each hemisphere of adult brain (Figure 2B) less than one day after emergence and also in the BR-SOG of pupa 5 days after incubation under LD 16:8 (Figure 3B). *Ap*5HTR_A_-ir was observed in the same cell bodies as *Ap*PTTH-ir in the BR-SOG of adult and early pupa (Figure 2C; Figure 3C). 

**Figure 4:**
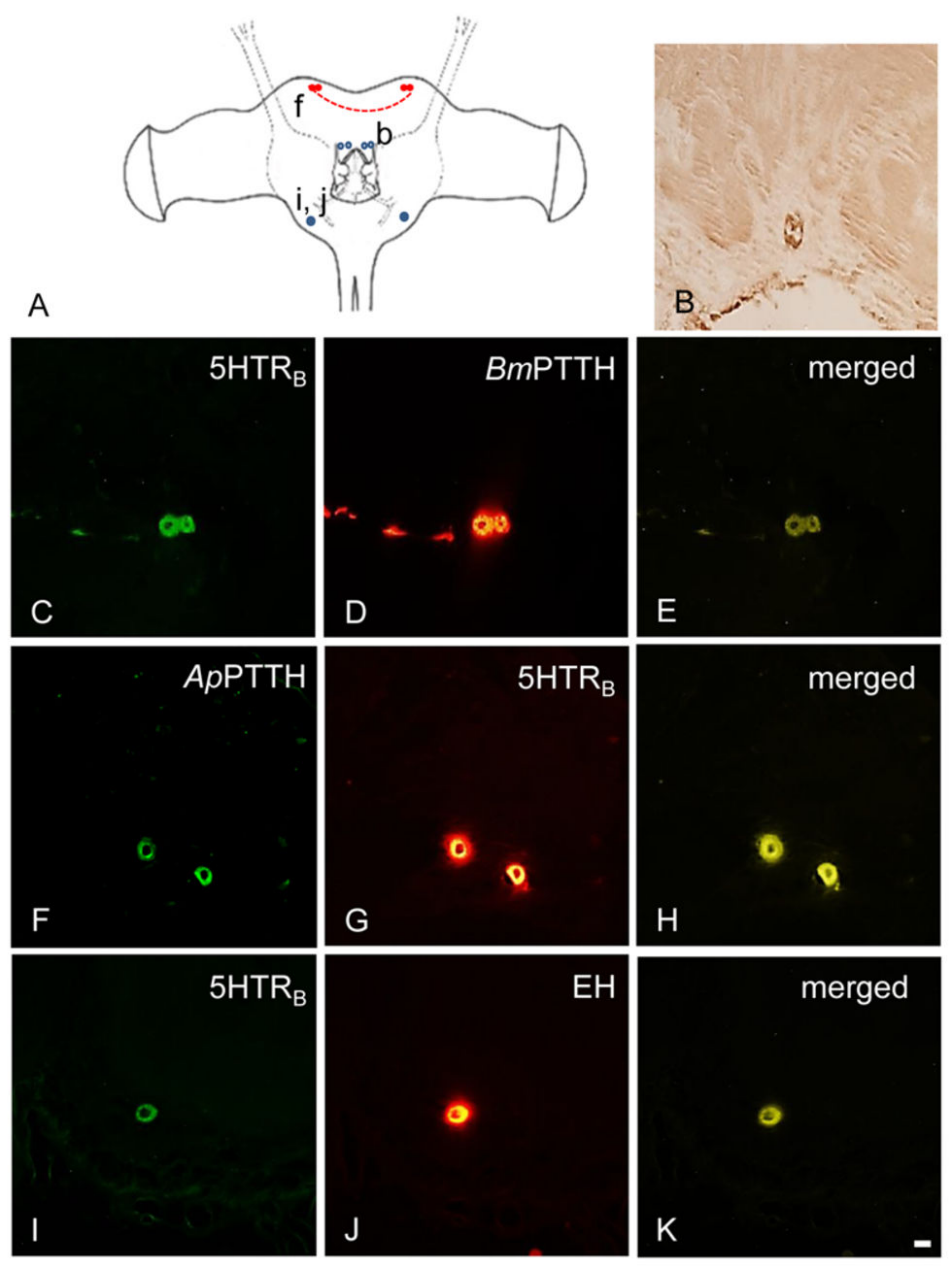
Colocalization of 5HTR_B_- and PTTH-ir in the adult BR-SOG of ***A. pernyi***. *Bm*PTTH-ir/*Ap*PTTH-ir in the DL region of protocerebrum of female adult less than one day old was co-localized with *Ap*5HTR_B_-ir (red filled circles). EH-ir in the adult brain of *A. pernyi* and its colocalization with *Ap*5HTR_B_-ir (blue filled circles) and unique distribution (open circles) of EH-ir in other regions of the brain. (A) The location of detected cells. Lower-case letters correspond to the regions shown in the photographs (e.g., b to B). (B) Two pairs *Bm*EH-ir cells in the DC region. (C, G) 5HTR_B_-ir in the DL region. (D) *Bm*PTTH-ir in the DL region. (E) Merged image of *Bm*PTTH-ir and 5HTR_B_-ir in the DL region. (F) *Ap*PTTH-ir in the DL region. (H) Merged image of *Ap*PTTH-ir and 5HTR_B_-ir in the DL region. (I) A 5HTR_B_-ir cell in the SOG. (J) One *Bm*EH-ir cell in the SOG. (K) Merged image of *Bm*EH-ir and 5HTR_B_-ir in the SOG region. Scale bar = 100 µm.

**Figure 5:**
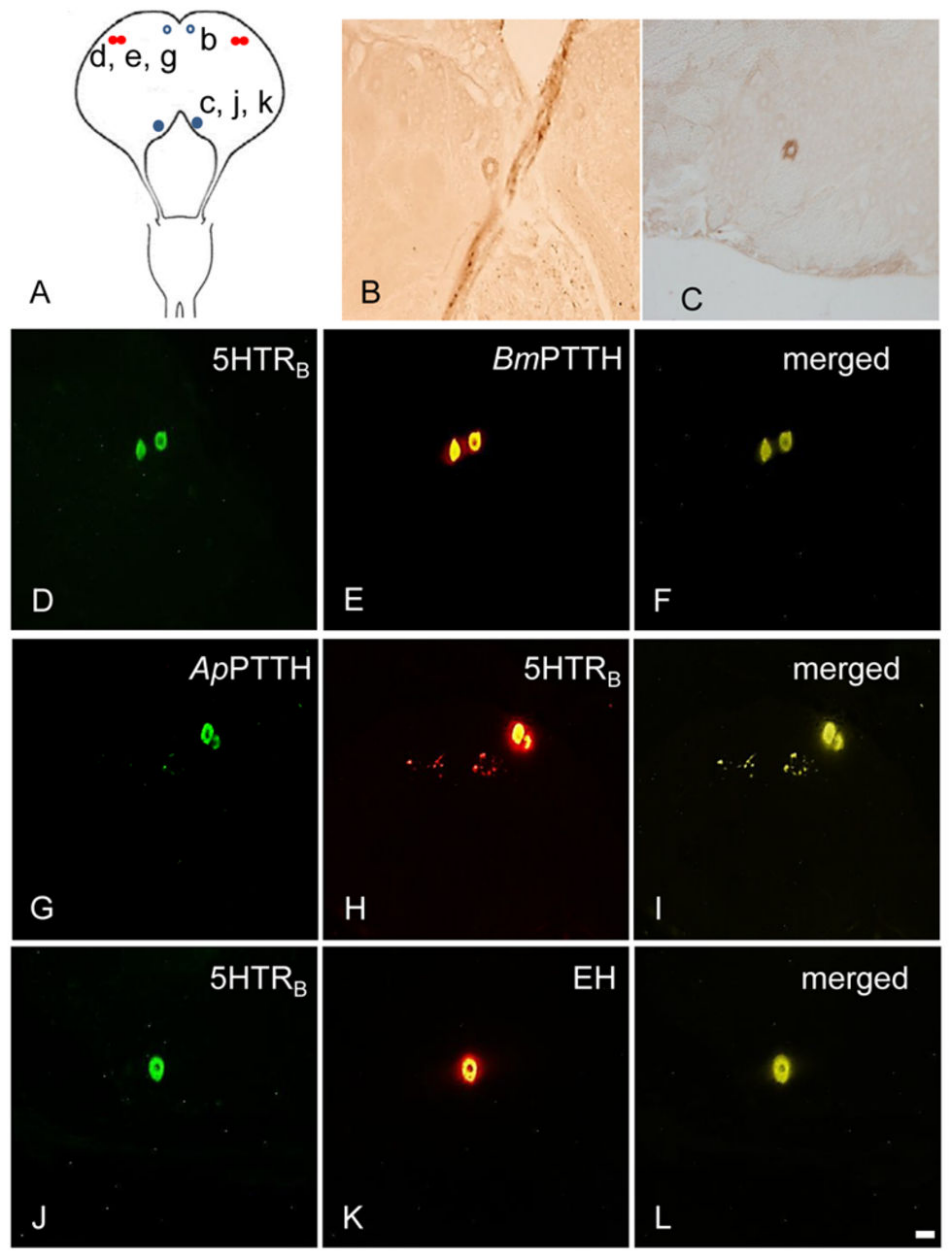
Colocalization of 5HTR_B_- and PTTH-ir in the erly pupal BR-SOG of ***A. pernyi***. *Bm*PTTH-ir/*Ap*PTTH-ir in the 5-day-old pupal brain of *A. pernyi* and its colocalization with *Ap*5HTR_B_-ir (red filled circles). *Bm*EH-ir in the 5-day-old pupa brain and its colocalization with *Ap*5HTR_B_-ir (blue filled circles) and unique distribution (open circles) of *Bm*EH-ir in other regions of the brain. (A) The loaction of detected cells. Lower-case letters correspond to the regions shown in the photographs (e.g., b to B). (B) One *Bm*EH-ir neuron in the PI. (C) *Bm*EH-ir in the DC region. (D, H) 5HTR_B_-ir in the DL region. (E) *Bm*PTTH-ir in the DL region. (F) Merged image of *Bm*PTTH-ir and 5HTR_B_-ir in the DL region. (G) *Ap*PTTH-ir in the DL region. (I) Merged image of *Ap*PTTH-ir and 5HTR_B_-ir in the DL region. (J) 5HTR_B_-ir in the DC region. (K) One EH-ir neuron in the DC. (L) Merged image of *Bm*EH-ir and 5HTR_B_-ir in the DC region. Scale bar = 100 µm.

*Ap*5HTR_B_-ir was also found in the DL of new adult and early pupa, as well as PTTH-ir cells. One pair of neurons were found in SOG of adult (Figure 4I) and in deutocerebrum (DC) of early pupa (Figure 5J). *Bm*PTTH-ir/*Ap*PTTH-ir and *Ap*5HTR_A_-ir/5HTR_B_-ir were co-localized in the DL of adult and pupa (Figure 2D-F, 2G-I; Figure 3D-F, 3G-I; Figure 4C-E, 4F-H; Figures 5D-F, 5G-I). 

Two pairs of large ns cells showed *Bm*EH-ir in the DC of adult (Figure 4B) and one pair did in the pars intercerebralis (PI) of pupal brain 5 days after incubation under LD 16:8 (Figure 5B), and a single cell did in DC per hemisphere (Figure 5C). This DC cell showed 5HTR_B_-ir. 5HTR_B_-ir and EH-ir were co-localized in the SOG of adult and in the DC of 5-day-old pupa (Figure 4I-K; Figure 5J-L), but 5HTR_A_-ir was not co-localized with EH-ir in the brain of adult and pupa. 

### Effects of RNAi against 5HTR_A_ and 5HTR_B_ on diapause

**Figure 6:**
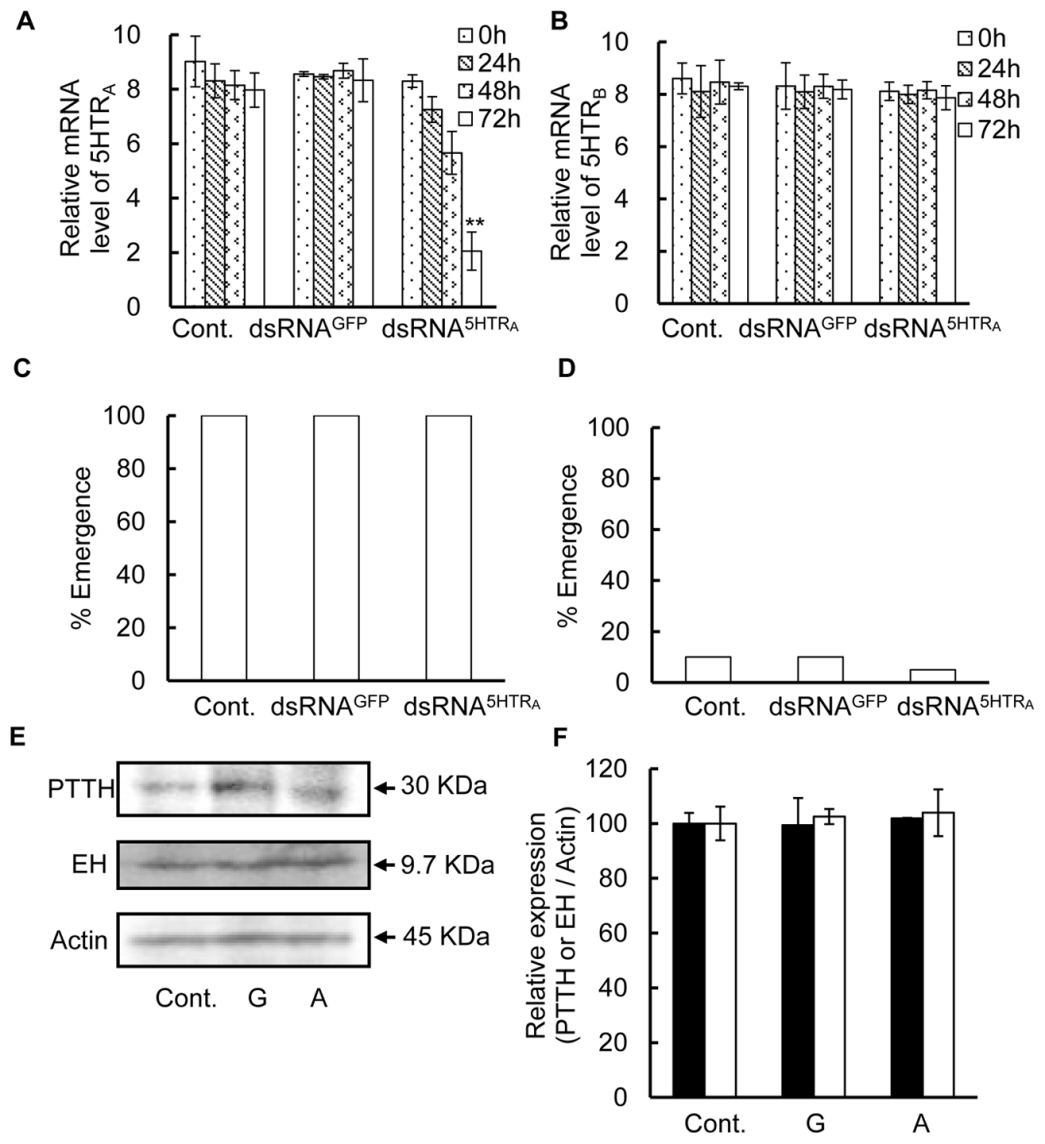
RNAi against 5HTR_A_ and the effect on photoperiodism. (A) Relative mRNA level of 5HTR_A_ in the BR-SOG of intact diapauses pupae (control) and those of pupae injected with dsRNA^GFP^ and with dsRNA^5HTRA^ were measured by q-PCR. The level of 5HTR_A_ mRNA was measured by qPCR with total RNA extracted from brains collected at 0, 24, 48 and 72 h under LD 16:8 at 25°C. (B) Relative mRNA level of 5HTR_B_ in the brain-SOG of the control pupae and those of pupae injected either with dsRNA^GFP^ or dsRNA^5HTRA^ under LD 16:8 at 25°C. (C) Adult emergence from the control, dsRNA^GFP^- or dsRNA^5HTRA^-injected diapause pupae under LD 16:8 at 25°C. (D) Adult emergence after injection of dsRNA to diapauses pupae targeting either at GFP or 5HTR_A_ under LD 12:12 at 25°C. Emergence was observed up to 40 days after injection. (E) Diapause pupae were injected with dsRNA^GFP^ and dsRNA^5HTRA^ or nuclease-free water as a control and 72 hours later the BR-SOG was dissected. *Ap*PTTH and *Bm*EH were separated by SDS-PAGE. The gel was subjected to western blot analysis. Equal amounts of total proteins from each group were loaded in each lane. A single band of around 30 kDa was detected with *Ap*PTTH antibody and a single band of around 9.7 kDa with *Bm*EH antibody. (F) The density of each band was quantified in relative to the value in the control. The filled columns, PTTH. Open columns, EH. Cont.: water was injection. G: dsRNA^GFP^ was injected. A: dsRNA^5HTRA^ was injected and pupae were kept under LD 12:12 at 25°C. The results are presented as the mean ± S.E.M. from three independent experiments and the differences were not significant from control by one-way ANOVA (Fisher's, LSD).

**Figure 7:**
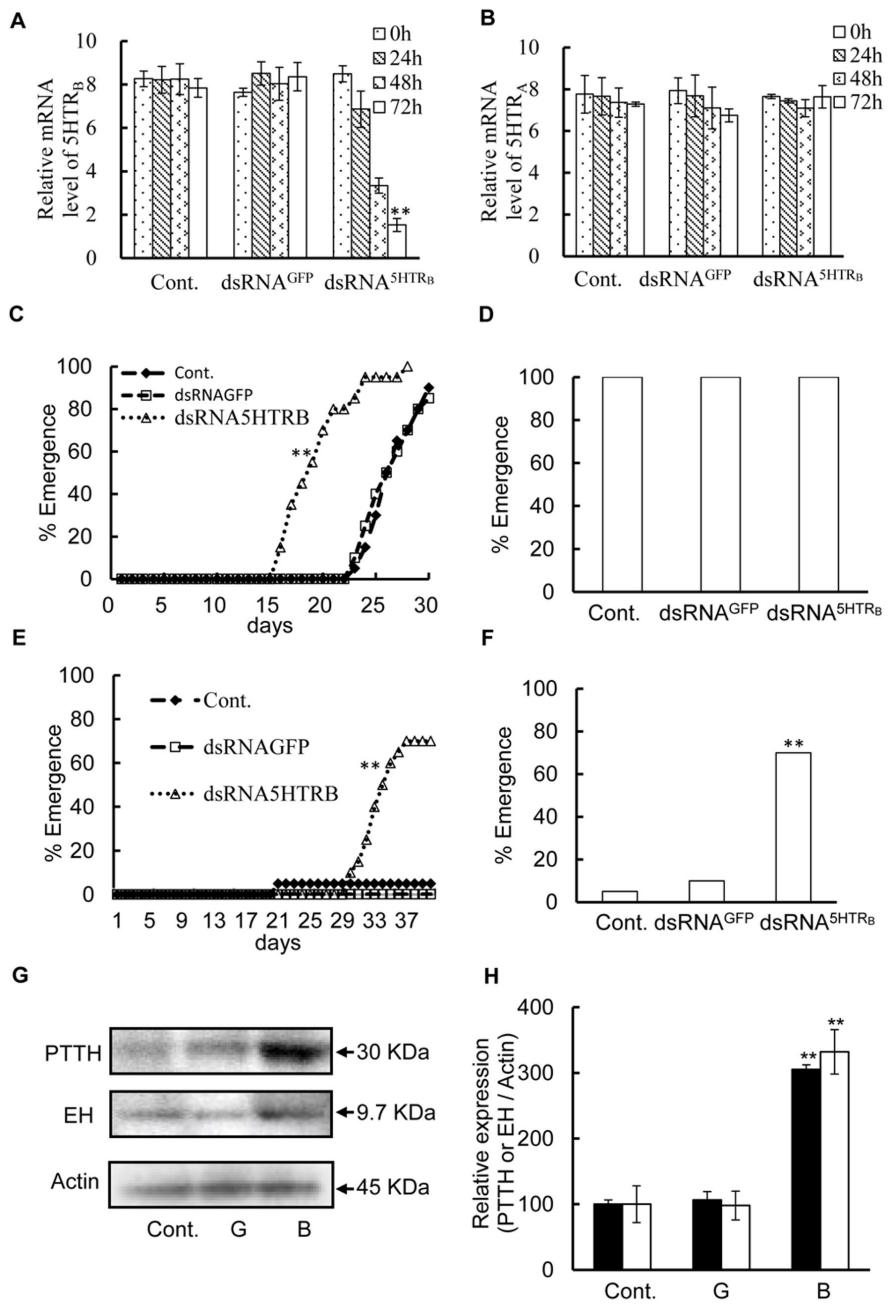
RNAi against 5HTR_B_ and the effect on photoperiodism. (A) mRNA level of 5HTR_B_ in the BR-SOG of diapause pupae (control), that of pupae injected with dsRNA^GFP^ and that with dsRNA^5HTRB^. The level of 5HTR_B_ mRNA was measured by q-PCR with total RNA extracted from BR-SOG 0, 24, 48 and 72 h after injection. (B) Relative mRNA level of 5HTR_A_ in the BR-SOG of diapause pupae (control), that of pupae injected with dsRNA^GFP^ and that with dsRNA^5HTRB^. (C) Adult emergence from the control, dsRNA^GFP^- and dsRNB^5HTRB^-injected diapauses pupae under LD 16:8 at 25°C. Asterisks indicate significant difference from control by Kaplan-Meier. ** *p*<0.01. (D) Adult emergence from the control, dsRNA^GFP^- and dsRNA^5HTRB^-injected pupae at 25°C under LD 16:8. Observation of emergence was continued up to 40 days after injection. (E) Diapause pupae were injected with dsRNA^GFP^ and dsRNA^5HTRB^ and adult emergence was recorded at 25°C under LD 12:12. Asterisks indicate significant difference from control by Kaplan-Meier. ** *p*<0.01. (F) Cumulatively 70% adults emerged in 40 days after injection dsRNA^5HTRB^. Asterisks indicate significant difference from control by Kaplan-Meier. ** *p*<0.01. (G) The expression of PTTH and EH was examined using western blot analysis 72 hours after the injection of dsRNA^GFP^ and dsRNA^5HTRB^, and nuclease-free water as a control. PTTH and EH were sepreated on SDS-PAGE. The gel was subjected to western blot analysis. Equal amounts of protein from each group were loaded on each lane. A single band of around 30 kDa was detected with *Ap*PTTH antibody and a single band of around 9.7 kDa with *Bm*EH antibody. (H) The densitometry of each band as the control was set to 100% after dsRNA^5HTRB^ injection. The filled columns, PTTH and open columns, EH. Cont.: water injection. G: dsRNA^GFP^ injection. A: dsRNA^5HTRB^ injection. The results are presented as the mean ± S.E.M. from three independent experiments. Asterisks indicate significant difference from control by one-way ANOVA (Fishers, LSD). ** *p*<0.01.

To identify the function of these receptors, diapause pupae were injected with dsRNA^GFP^, dsRNA^5HTRA^ or dsRNA^5HTRB^ and then kept under LD 16:8 or LD 12:12 at 25°C. The levels of 5HTR_A_ and 5HTR_B_ mRNAs were significantly lower after 72 hours of injections of dsRNA^5HTRA^ and dsRNA^5HTRB^ (Figures 6A, 7A), respectively, while the injection of dsRNA^5HTRA^ or dsRNA^5HTRB^ did not alter the level of 5HTR_B_ mRNA and 5HTR_A_ mRNA, respectively (Figures 6B, 7B). The injection of dsRNA^GFP^ had no effect on the transcription level of both receptor genes. Therefore, it was concluded that RNAi acted specifically. The adult emergence from pupae injected with dsRNA^5HTRA^ was the same as that of the control groups, that is, uninjected and dsRNA^GFP^-injected under LD 16:8 and LD 12:12 (Figure 6C, D). However, adults emerged from pupae that were injected with dsRNA^5HTRB^ earlier than the control groups under LD 16:8 (Figure 7C, D), and the injection of dsRNA^5HTRB^ terminated pupal diapause even under LD 12:12 (Figure 7E, F). 

Seventy-two hours after dsRNA^5HTRA^ or dsRNA^5HTRB^ injections, PTTH- and EH-ir bands detected on western blots were examined. No change was detected in the samples treated with dsRNA^5HTRA^ from uninjected and dsRNA^GFP^-injected controls (Figure 6E, F), while a significant increase was observed in the dsRNA^5HTRB^-treated group (Figure 7G, H). A single band of around 30 kDa was detected in western blotting with *Ap*PTTH antibody, which had a molecular mass close to that of the predicted size of PTTH of *A. pernyi* (GenBank: AAB05259.1, 221aa=24.56 kDa). A single band of around 9.7 kDa detected with *Bm*EH antibody was close to the estimated values of the EH of *M. sexta* (GenBank: AAA29311.1, 88aa=9.67 kDa) and *B. mori* (GenBank: AAA29310.1, 88aa=9.67 kDa). dsRNA^5HTRB^ intensified these bands by more than 300% (Figure 7G, H). This result suggests that 5HTR_B_ not only controls the release but the synthesis of PTTH and EH. 

### Effect of 5HT pharmacology on diapause determination

**Figure 8:**
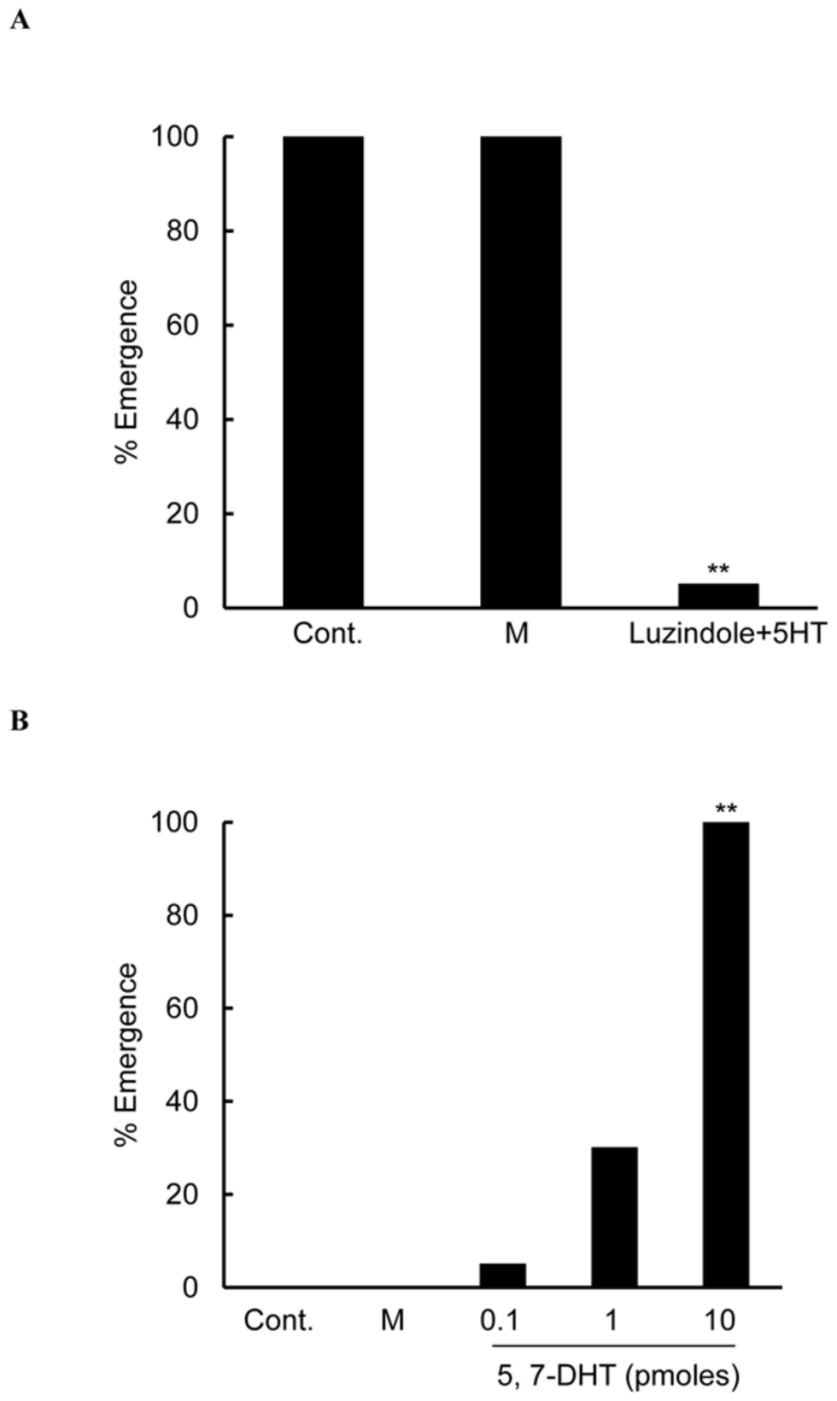
Pharmacological confirmation of RNAi effect targeting at 5HTR_B_. Effect of injections of 5HT, 5,7-DHT and luzindole on photoperiodism. (A) Diapause pupae were injected either with 5 µl water and 5 µl DMSO (Mock injection) or 5 pmoles Luzindole plus 5 pmoles 5HT in the same volume of solvent and placed under LD: 16:8 at 25°C. Cumulatively 5% adults emerged in 40 days after injection. Cont.: untreated. M: injection with distilled water and DMSO. Luzindole +5HT: luzindole and 5HT co-injected. (B) Diapause pupae were injected with 5,7-DHT at three doses and thereafter the pupae were kept under LD 12:12 at 25°C. Cont.: untreated. M: mock injection with 10 µl distilled water. 5,7-DHT: injected with 5,7-DHT dissolved in the same volume as in the mock. 5 percentage points, 30 percentage points and 100 percentage points adult emergence was observed in 40 days. Asterisks indicate significant difference from control by Kaplan-Meier. ** *p*<0.01.

We have shown that insect aaNAT is involved in the circadian regulation of photoperiodic termination of regulation of pupal diapause in *A. pernyi* (under submission). A melatonin receptor antagonist, luzindole, inhibited the release of PTTH in cockroach [14]. First, we did single injections of 5HT and luzindole. After injection, the time of emergence was delayed in both groups (data unpublished). Since NAT metabolizes 5HT to melatonin via *N*-acetylserotonin and the two terminal amines have opposite physiological functions, we made a double injection of 5HT and luzindole. Effect of 5HT should thereby most properly evaluated, since we cannot control NAT activity. When NAT activity is high, the injected 5HT should be converted promptly to melatonin that should antagonist 5HT effect. We showed that the co-injection of 5 pmoles 5HT and luzindole delayed diapause termination under LD 16:8 (Figure 8A). This suggests a dual mechanism of diapause. 5HT maintains diapause while melatonin terminates it. This notion is further supported by the injection of 10 pmoles 5,7-DHT into diapause pupae, which induced emergence in a dose-dependent manner under LD 12:12 (Figure 8B).

## Discussion

This study focused on the roles of 5HTRs in photoperiodism that regulates pupal diapause in *A. pernyi*. 5HTRs have been investigated in many insects, including *Drosophila melanogaster* [28], *Apis mellifera* [29], two crickets (*Dianemobius. nigrofasciatus* and *Allonemobius. allardi*) [25] and *M. sexta* [8]. We have cloned two types of 5HTR in *A. pernyi*, showing that only 5HTR_B_ transcription was under circadian influence [24]. We then examined the distributions of 5HTR-ir in the BR-SOG of *A. pernyi*. The two receptors have both shared and unique distributions: 5HTR_A_-ir and 5HTR_B_-ir were shared in the PTTH-ir neurosecretory cells but only 5HTR_B_ was colocalized with EH-ir, which was therefore unique.

*A. pernyi* overwinters in pupal diapause under short-day conditions, while it produces another generation under long-day conditions. Developmental switch must be controlled by the release or inhibition of release of PTTH. Once diapause is initiated, it is terminated by ten cycles of long days or low temperature for several months [30]. 

PTTH-ir has been immunohistochemically investigated in some insects, including *B. mori* [12], *A. pernyi* [6], *M. sexta* [13] and *P. americana* [26]. 5HT stimulated PTTH release *in vitro* also in a BR-SOG culture co-incubated with prothoracic gland in *B. mori* [31], but 5HTR subtypes have not been characterized in this species. Since 5HT is metabolized to melatonin, which of the indoleamines stimulates PTTH release cannot be determined. Richer et al. [17] have demonstrated that, in *P. americana*, melatonin stimulates PTTH release *in vitro*, whereas 5HT inhibits this release. 

 We here demonstrated that *Bm*PTTH-ir/*Ap*PTTH-ir ns cells at DL of both adult and early pupa of *A. pernyi* was co-localized with 5HTR-ir. Two types of *Ap*5HTR-ir have also been investigated in two ground crickets, *D. nigrofasciatus* and *A. allardi*, where 5HTR_A_-ir was located in the PI, DL, optic tract, optic lobe and midline of SOG, whereas 5HTR_B_-ir was located in the PI, DL and weakly in the optic lobe, tritocerebrum and midline of SOG in both crickets. In *A. allardi*, both receptors may be involved in circadian photo-entrainment or photoperiodism because they were co-localized with CLK-ir; in *D. nigrofasciatus*, only 5HTR_B_ was co-localized with CLK-ir. Therefore, it may be involved in circadian photo-entrainment or photoperiodism [25]. In *D. melanogaster*, *Dm*5HTR_1B_ is expressed in clock neurons, and changed the molecular and behavioral responses of the clock to light [32]. This effect of 5HT is mediated via Dm5HTR_1B_, but not *Dm*5HTR_1A_. These results showed that *Dm*5HTR_1A_ and *Dm*5HTR_1B_ play different roles [33]. *Dm*5HTR_1A_ was modulated in the larval response to light [34]. *Dm*5HTR_1A_ also regulates sleep, learning and memory [33]. In our results, the function of *Ap*5HTR_A_ remains unresolved in relation to the photoperiodic regulation of diapause, but the possibility remains that it regulates PTTH release or synthesis via routes other than the photoperiodic pathway. It may be involved in the inhibition of activation by temperature. *Am*5HT_1A_ was shown to be involved in the regulation of honeybee phototactic behavior [29]. It also affects olfactory learning in the honeybee [35]. 5HTR_A_ may be involved in the regulation of PTTH release in *A. pernyi*, but not via the photic route, because the mRNA level did not react to long-day activation. Another possibility is that it regulates bilateral coupling with contralateral PTTH ns cells, since PTTH-ir fibers can be traced to the contralateral ns cells. 

These data strongly suggest that melatonin and a related indolamine play a key role in the release of PTTH [17]. Then, the next question to ask should be about what locks up the release of PTTH to initiate/maintain diapause. The answer is 5HT/5HTR_B_ binding. The expression of 5HTR_B_ showing circadian fluctuation in mRNA [24] is photoperiodically controlled and the injection of dsRNA^5HTRB^ accelerated diapause termination even under LD 12:12. This notion was supported pharmacologically. After the melatonin receptor was shut down, 5HT injection inhibited diapause termination even under LD 16:8. The injection of 5,7-DHT, terminated diapause in a dose-dependent manner under LD 12:12. This poison may destroy both 5HT and melatonin. However, a significant effect of its injection was early termination of diapause even under LD 12:12. The results indicate that without melatonin activation the depletion of 5HT terminates diapause.

Because not only dsRNA^5HTRB^ injection resulted in the same response but also increased PTTH production, diapause is not simply a default condition of PTTH release but enhanced by 5HT/5HTR mechanism particularly in the site of PTTH synthesis.

**Figure 9:**
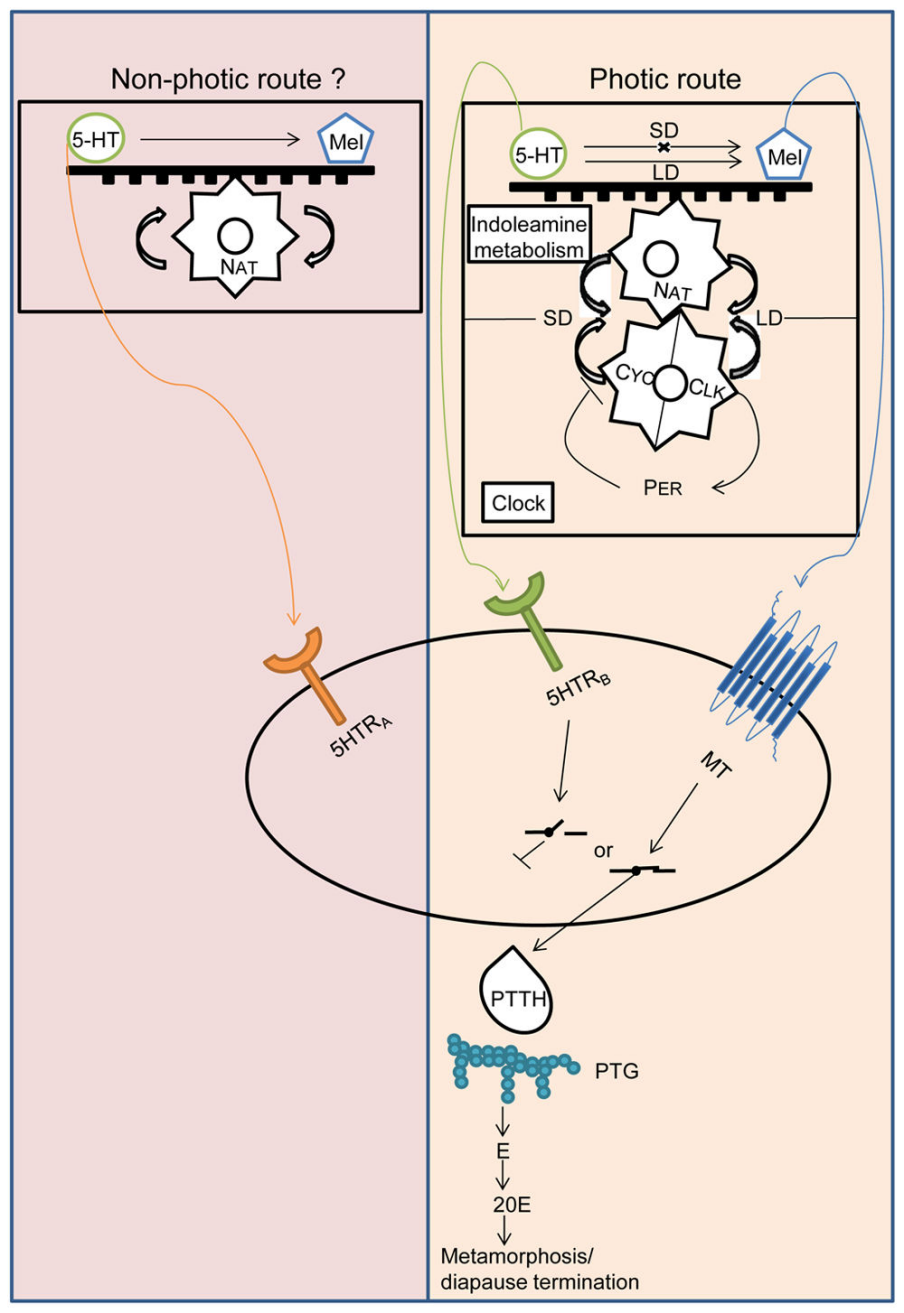
Schematic illustration of 5HTRs role on diapause induction/maintenance in pupal diapause of *A. pernyi*. The moth has two 5HTR subtypes, 5HTR_A_ and 5HTR_B_.The former subtype shows no transcription rhythm [24] and did not respond to photoperiodic activation by long day, while the latter showed rhythmic expression and responded to photoperiodic activation. Therefore, it may be regulated by circadian system. Via one type of arylalkylamine N-acetyltransferase, aaNAT. This engyme is encoded by *nat* is that a circadian-controlled gene (ccg) since dsRNA^CYC^, and dsRNA^CLK^ suppressed *nat* transcription and dsRNA^NAT^ dysfunctioned photoperiodism. Transcription of 5HTR_A_ was not rhythmic. MT-binding closes the endocrine switch to PTTH release that finally terminates diapause, while 5HTR_B_ cuts this circuit to enforce or initiate diapause. Diapause of *A.penyi* is therefore under binary regulation and circadian system regulates at least two points in this system, *nat* transcription and 5HTR_B_ expression. aaNAT, arylalkylamine N-acetyltransferase. PER, Period protein, a negative regulator of transcription translation feedback. CYC/CLK, heterodimeric circadian transcription regulator. Mel, melatonin. MT, melatonin receptor. LD, long day. SD, short day. PTTH, prothoracicotropic hormone. PTG, prothoracic gland. E, ecdysone. 20E, 20 hydroxyecdysone.

Figure 9 is a projected over-all view of dual regulation mechanism of pupal diapause in *A. pernyi*. Photoperiodic/circadian gear affects aaNAT via circadian transcription factors, CYC and CLK (photic route). If 5HT is overproduced, diapause is initiated and maintained via 5HTR_B_. If melatonin is overproduced, it activates MT that stimulates PTTH release. A balance between the two indoleamines is regulated at NAT. Non-photic environmental condition such as low temperature may inactivate 5HTR_A_. 

At the end of molting, EH release responds to circadian gate and a 20E decline [36]. EH-ir has been investigated in many insects, including *Siphlonurus armatus* [37], *M. sexta* [38] and *B. mori* [39]. 5HTR_B_ is not only involved in PTTH release/synthesis but also in EH release/synthesis. We have demonstrated co-localization of EH-ir and 5HTR_B_-ir. The fact that the injection of dsRNA^5HTRB^ increased the EH synthesis/accumulation suggests that 5HTR_B_ is involved in EH synthesis since EH release is not leaky but gated. 

## Conclusions

The role of 5HTR_B_ in the diapause induction/maintenance mechanism in the brain of *A. pernyi* is to lock the gate for PTTH release, because dsRNA^5HTRB^ opened the gate. Photoperiods therefore affect two ways 1) by changing relative abundance of 5HT and melatonin via circadian regulation of aaNAT and 2) by changing 5HTR_B_ expression via circadian system, since 5HTR_B_ showed a day/night fluctuation and responded to long-day activation. We still do not know photoperiodic/circadian influence over MT. This is our next task. Since 5,7-DHT treatment induced early emergence under LD 12:12, diapause is not only regulated by the release of PTTH but the main mechanism to induce/maintain diapause is 5HTR_B_ mechanism. This drug poisons both 5HT and melatonin. If melatonin is the only regulator of PTTH release, the injection would not induce early emergence under LD 12:12.

## Supporting Information

Table S1 **Data of primary antibodies used in this study.**
(DOCX)
