# Supplementary material for: Serotonin Receptor B May Lock the Gate of PTTH Release/Synthesis in the Chinese Silk Moth, Antheraea pernyi; A Diapause Initiation/Maintenance Mechanism?
Source: PLoS One. 2013 Nov 4;8(11):e79381. doi: 10.1371/journal.pone.0079381 (PMC3817057; doi:10.1371/journal.pone.0079381)
Supplement: Table S1 — Data of primary antibodies used in this study. (DOCX) [file pone.0079381.s001.docx]

**Supplemental information**

**Table S1.** Data of primary antibodies used in this study

| Antibody used | Immunized Animal | Working Dilution | Source |
| --- | --- | --- | --- |
| *Ap*5HTR_A_ and _B_ | Rabbit | 1:1000 | M. Takeda, Kobe University, Japan |
| *Bm*EH | Rat | 1:2000 | Purchased (Santa Cruz; at-34973) |
| *Ap*PTTH | Rabbit | 1:2000 | Sauman and Reppert, 1996 |
| *Bm*PTTH | Rat | 1:1500 | M. Takeda, Kobe University, Japan |
